# PpiD is a player in the network of periplasmic chaperones in *Escherichia coli*

**DOI:** 10.1186/1471-2180-10-251

**Published:** 2010-09-29

**Authors:** Yvonne Matern, Birgitta Barion, Susanne Behrens-Kneip

**Affiliations:** 1 Abteilung Molekulare Genetik und Präparative Molekularbiologie, Institut für Mikrobiologie und Genetik, Georg-August-Universität Göttingen, Grisebachstr. 8, D-37077 Göttingen, Germany; 2 Robert Koch-Institut, Nordufer 20, D-13353 Berlin, Germany

## Abstract

**Background:**

The inner membrane-anchored periplasmic folding factor PpiD is described as a parvulin-like peptidyl prolyl isomerase (PPIase) that assists in the maturation of the major beta-barrel outer membrane proteins (OMPs) of *Escherichia coli*. More recent work however, calls these findings into question. Here, we re-examined the role of PpiD in the *E. coli *periplasm by analyzing its functional interplay with other folding factors that influence OMP maturation as well as general protein folding in the periplasmic compartment of the cell, such as SurA, Skp, and DegP.

**Results:**

The analysis of the effects of both deletion and overexpression of *ppiD *on cell envelope phenotypes revealed that PpiD in contrast to prior observations plays only a minor role, if any, in the maturation of OMPs and cannot compensate for the lack of SurA in the periplasm. On the other hand, our results show that overproduction of PpiD rescues a *surA skp *double mutant from lethality. In the presence of increased PpiD levels *surA skp *cells show reduced activities of both the SigmaE-dependent and the Cpx envelope stress responses, and contain increased amounts of folded species of the major OMP OmpA. These effects require the anchoring of PpiD in the inner membrane but are independent of its parvulin-like PPIase domain. Moreover, a PpiD protein lacking the PPIase domain also complements the growth defects of an *fkpA ppiD surA *triple PPIase mutant and exhibits chaperone activity *in vitro*. In addition, PpiD appears to collaborate with DegP, as deletion of *ppiD *confers a temperature-dependent conditional synthetic phenotype in a *degP *mutant.

**Conclusions:**

This study provides first direct evidence that PpiD functions as a chaperone and contributes to the network of periplasmic chaperone activities without being specifically involved in OMP maturation. Consistent with previous work, our data support a model in which the chaperone function of PpiD is used to aid in the early periplasmic folding of many newly translocated proteins.

## Background

It is well established that numerous chaperones, folding catalysts and proteases exist in the periplasm of *E. coli *and cooperate in protein folding and protein quality control in this cellular compartment of the cell. At least three of these factors, SurA, Skp and DegP, assist in the maturation of integral β-barrel outer membrane proteins (OMPs), a major class of proteins in the *E. coli *outer membrane, and are thought to be responsible for the transport of OMP folding intermediates through the periplasm to the OMP assembly site, a multi-protein complex in the outer membrane [1].

The chaperone and peptidyl-prolyl isomerase (PPIase) SurA is specialized for the biogenesis of OMPs. SurA preferentially interacts with newly-synthesized OMPs *in vitro *[2] by specifically recognizing and binding to peptide sequences that are characteristic of OMPs [3,4]. Only a subset of OMPs however, appears to directly depend on SurA for maturation [5]. The two biochemical activities of SurA reside in distinct regions of the protein [2]. The PPIase activity is carried in the second of two iterative parvulin-like domains (domain I and domain II) located in the C-terminal half of the protein [2,6]. The chaperone activity, which is required and sufficient for the so far known biological role of SurA, is contained in a module formed by its N-terminal region and a short C-terminal sequence [2]. Lack of SurA gives phenotypes that are indicative of disturbances in OMP biogenesis and of a defective cell envelope. Such phenotypes are reduced levels of the major OMPs OmpA, LamB, and OmpC in the outer membranes of the cells, increased sensitivity to hydrophobic agents, such as SDS/EDTA, bile salts, and the antibiotic novobiocin, and a constitutively induced σ^E^-dependent envelope stress response [6-8]. The σ^E ^pathway together with the Cpx signal transduction pathway monitors and controls the protein folding state in the cell envelope [9].

The small periplasmic chaperone Skp and the protease-chaperone DegP affect general protein folding in the periplasm and assist in the biogenesis of OMPs. A *skp *mutant shows phenotypes that are similar to but less severe than those of a *surA *mutant [7]. Moreover, deletion of *skp *confers synthetic lethality in a *surA *mutant, as does deletion of *degP *[2,10]. *degP skp *double mutants on the other hand are viable. It has therefore been proposed that DegP and Skp act together in one pathway of OMP maturation whereas SurA acts in a separate parallel pathway [5,10]. Skp has been shown to interact with early OMP folding intermediates at the periplasmic side of the inner membrane [11,12] and to keep immature OMPs in a soluble state [13,14]. DegP on the other hand, was found to bind to and stabilize folded OMP monomers [15] and thus appears to act downstream of Skp in the proposed Skp/DegP pathway for OMP maturation.

Conflicting results have been reported regarding the involvement of the periplasmic PpiD protein in the biogenesis of OMPs. PpiD is anchored to the inner membrane by an N-terminal transmembrane segment and consists of a single parvulin domain flanked by large N- and C-terminal protein regions. The N-terminal region shares sequence similarity with the N-terminal region of SurA, which comprises the major part of the SurA chaperone module ([16-19]; see additional file 1). Several previous findings suggested that PpiD and SurA have overlapping functions in OMP biogenesis [18]. First, a *ppiD *mutant was documented to have phenotypes that are similar to those of a *surA *mutant and are suppressed by multicopy *surA*. Second, the simultaneous deletion of *ppiD *and *surA *was reported to cause lethality. More recently however, *surA ppiD *mutants were shown to display no visible growth defects [20]. Finally and most importantly, *ppiD *was isolated as a multicopy suppressor in a *surA *mutant. Remarkably however, whereas the *surA *phenotypes result from loss of chaperone function [2], a high PPIase activity of PpiD was identified as the complementing biochemical activity [18]. Most recently, this result was disputed by the finding that the isolated parvulin domain of PpiD is devoid of detectable PPIase activity [19]. Here, we analyzed the functional interplay of PpiD with SurA, Skp, and DegP to define its role in the *E. coli *periplasm.

## Results

### Re-examination of PpiD function in the biogenesis of OMPs

To resurvey the role of PpiD in OMP maturation we analyzed the physiological consequences of both inactivation and overexpression of *ppiD *in wild-type cells and in the *surA *and *skp *mutants, respectively, using phenotypes known to report on OMP biogenesis and outer membrane integrity, such as σ^E ^activity, resistance of the cells to SDS/EDTA and to the antibiotic novobiocin, as well as the levels of major OMPs in their outer membranes. In contrast to previous work [18] we found that expression of multicopy *ppiD *from the IPTG-inducible *P*_trc _promoter does not suppress the *surA *mutant phenotypes but rather interferes with cell growth (data not shown). We therefore used a plasmid (pPpiD) that carries *ppiD *under control of its natural promoter, which is positively regulated by the classical cytoplasmic σ^32^-dependent heat-shock response and by the Cpx two-component system [18,21].

**Figure 1:**
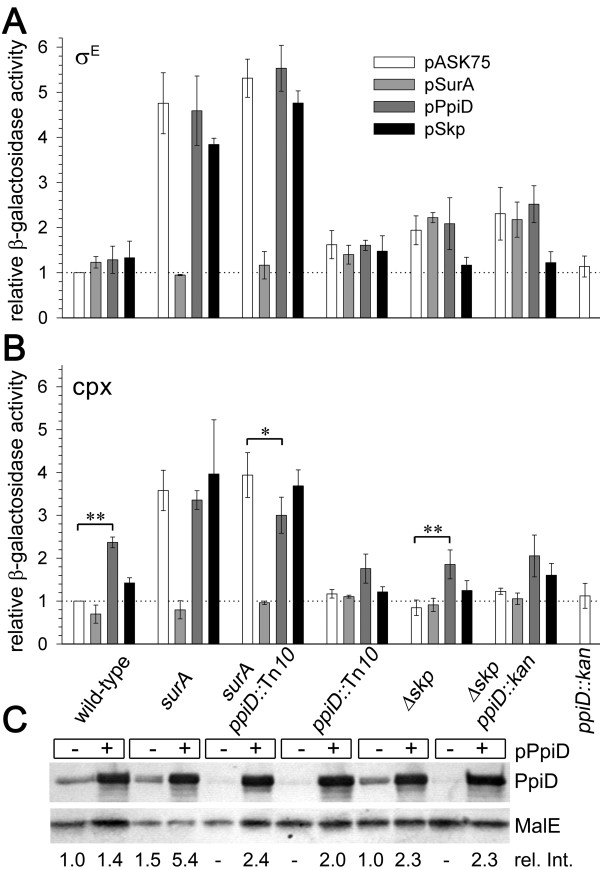
**Response of the σ^E^-dependent and the CpxA/R-regulated envelope stress pathways to inactivation and overexpression of *ppiD***. Eσ^E ^(A) and Cpx (B) activities in the indicated strains carrying SurA (light gray bars), PpiD (dark gray bars), and Skp (black bars) encoding plasmids or an empty vector (pASK75; white bars) were assayed by monitoring the accumulation of β-galactosidase resulting from σ^E^-dependent *rpoH*P3::*lacZ *and from Cpx-meditated *cpxP*-lacZ reporter expression, respectively. Cells were grown in LB (σ^E^) or in LB buffered at pH 7.0 (Cpx) at 37°C and β-galactosidase activities were determined as described in *Methods *and compared to that of wild-type cells. Results represent the average of at least two independent experiments (*P ≤ 0.05; **P ≤ 0.01 Student's t-test). Qualitatively similar results were obtained from cells grown at 30°C (data not shown). (C) Western blot analysis of crude extracts derived from cells with (+) and without (-) pPpiD. A volume of sample equivalent to 4 × 10^7 ^cells was loaded onto each lane. The anti-PpiD antiserum showed a weak unspecific cross-reaction with a similar sized unknown protein. The intensity of the PpiD signal relative to that in the wild-type strain (rel. Int.) was calculated using MalE as the internal standard for each lane.

Consistent with recent observations [20], the inactivation of *ppiD *in a *surA *strain did not cause lethality. Also, neither the inactivation nor the overexpression of *ppiD *in the tested strains had major effects on the activity of σ^E ^(Figure 1), on the plating efficiencies on 0.5% SDS/0.5 mM EDTA (Table 1), on the sensitivities of the cells to novobiocin, or on the levels of major OMPs in their outer membranes (data not shown). However, as is also the case for strains lacking Skp, PpiD-deficient strains showed slightly retarded growth on plates containing 0.5% SDS and 0.5 mM EDTA. At increased concentrations of SDS (2%) a *ppiD skp *double mutant even revealed a small (3-to 4-fold) plating defect (Table 1), but showed no major changes in the activity of σ^E ^and in the amounts of OMPs in the outer membranes of the cells relative to the *Δskp *single mutant (Figure 1 and data not shown). Thus, loss of PpiD appears to slightly interfere with outer membrane integrity without notably affecting the assembly of OMPs. Together these results suggest that PpiD plays only a minor role, if any, in the biogenesis of OMPs in the strain background used here.

**Table 1:** Plating efficiencies on SDS/EDTA

Strain	Plasmid	Efficiency of plating^a ^on 0.5 mM EDTA	
		
		+ 0.5% SDS	+ 2% SDS
wild-type	None	0.90	0.54 ± 0.146
	pASK75	0.93 ± 0.061	

*surA*	pASK75^b^	8.0, 0.028, and 0.011 [× 10^-3^]	
	pSurA	1.0 ± 0.13	
	pPpiD^b^	5.8, 0.011, and 0.032 [× 10^-3^]	

*ppiD*::Tn*10*	None		0.66 ± 0.156
	pASK75	0.96 ± 0.087	

*ppiD*::*kan*	None		0.42 ± 0.184
	pASK75	0.81 ± 0.067	

*surA ppiD*::Tn*10*	pASK75^b^	2.6, 7.2, and 0.66 [× 10^-3^]	.
	pSurA	0.7 ± 0.02	
	pPpiD^b^	4.1, 2.6, and 0.25 [× 10^-3^]	

*Δskp*	None	0.87 ± 0.02	0.57 ± 0.042
	pQE60	1.04	

*Δskp ppiD*::*kan*	none	1.01 ± 0.06	0.17 ± 0.042
	pQE60	1.0	

^a^Values are the averages of at least three independent experiments.

^b^The plating efficiencies of three independent experiments are shown, as the wide range of variation did not allow averaging. Larger variations in the efficiencies of plating were observed only for strains showing strongly increased SDS/EDTA sensitivity and likely result from minor fluctuations in the concentration of these membrane perturbants in the different batches of medium (prepared freshly for each experiment).

### Effects of inactivation and overexpression of *ppiD *on the Cpx envelope stress response

The σ^E ^signal transduction pathway partially overlaps with the CpxA/R pathway in sensing and responding to folding stress in the cell envelope [9]. Since *ppiD *is a member of the Cpx regulon [18] we asked whether the Cpx system would respond to inactivation or increased expression of *ppiD*. As shown in Figure 1B, inactivation of *ppiD *had no significant effect on Cpx activity in any of the tested strains, indicating that PpiD is not specifically involved in cell envelope functions that are monitored by the Cpx stress response pathway. In contrast, lack of SurA induced the Cpx response ~4-fold, as is consistent with the involvement of SurA in OMP and pilus biogenesis [20] and with misfolding pilus subunits being sensed by the Cpx signaling system [22]. The presence of *ppiD *in multicopy led to an about 2-fold induction of the Cpx response in all strains but the *surA *single and the *surA ppiD *double mutants. In the *surA ppiD *double mutant increased expression of *ppiD *from pPpiD slightly reduced Cpx activity, whereas it showed no significant effect on Cpx activity in the *surA *single mutant.

### *ppiD *is a multicopy suppressor of the lethal *surA skp *phenotype

**Figure 2:**
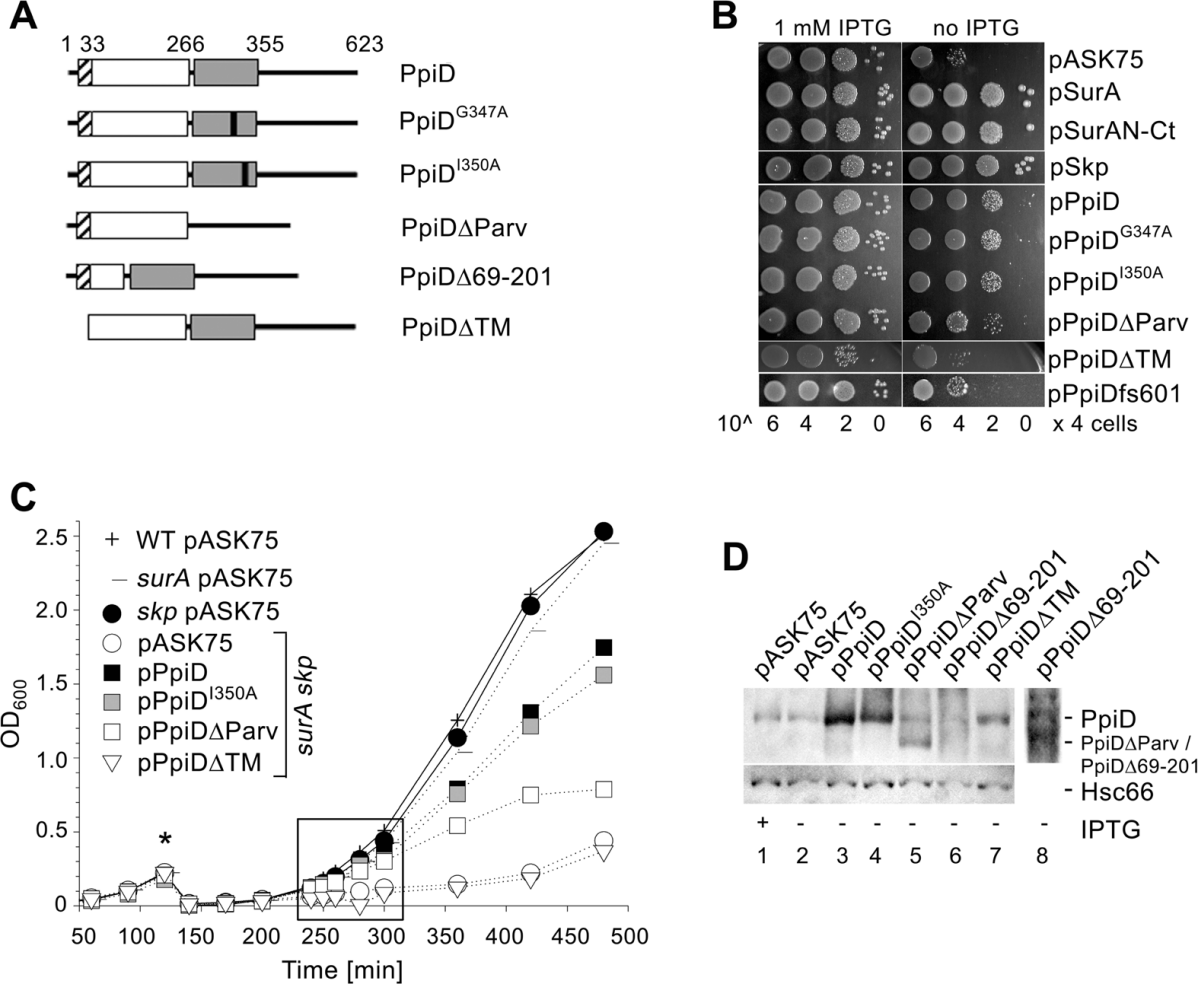
**Suppression of the lethal phenotype of *surA skp *cells by multicopy *ppiD***. (A) Schematic representation of PpiD and its variants used in this study, with amino acid residues numbered as in the full-length PpiD polypeptide. Diagonally striped box: transmembrane segment; white box: N-terminal region; Gray box: parvulin domain, with alanine substitutions indicated by black bars. PpiDΔTM was preceded by the SurA signal peptide so that it would be secreted into the periplasm (see *Methods*). (B) Growth of the SurA-depletion strain *P_Llac-O1_*-*surA Δskp *(SB44452) carrying pASK75 (empty vector), pSurA, pSkp, and pPpiD, respectively. Cells were grown overnight in the presence of IPTG, after dilution spotted on LB plates ± IPTG, and incubated at 37°C for 16-24 h. (C) Growth of the strains *P_Llac-O1_*-*surA *(SB44454) and *P_Llac-O1_*-*surA Δskp *(SB44452) at 37°C in liquid LB with (solid lines) and without (dotted lines) IPTG, resulting in the indicated "genotypes" wild-type (WT), *surA*, *skp*, and *surA skp*. The asterisk marks the point of sub-culturing (see *Methods*). Within the framed interval samples were taken for further analysis. Note that the *Δskp surA *strain containing pASK75 or pPpiDΔTM resumed growth after ~360-minute cultivation without IPTG. Western blotting revealed that at this point the cells also resumed production of SurA (see additional file 3). In contrast, SurA levels remained low in *Δskp surA *pPpiD cells during the entire course of growth, indicating that increased PpiD levels compensate for the simultaneous lack of SurA and Skp. (D) PpiD proteins in *P_Llac-O1_*-*surA Δskp *cells after 240-minute growth in LB without IPTG. Extracts from 4 × 10^7 ^cells were loaded in each lane and analyzed by western blotting. Lane 8 shows lane 6 after prolonged development of the blot to visualize the protein. Cytoplasmic Hsc66 served as a loading control. Data for one representative experiment are shown.

We also asked whether *ppiD *in multicopy would suppress the synthetic lethality of a *surA skp *mutant. SurA-depletion strains were constructed by placing the chromosomal *surA *gene under the control of the IPTG-inducible promoter *P_Llac-O1 _*[23], so that expression of *surA *could be shut off in the absence of IPTG. As expected, *P_Llac-O1_*-*surA Δskp *cells grew poorly without IPTG but normal growth was restored by providing copies of either *surA *or *skp *on a plasmid (Figure 2B). Unexpectedly, growth in the absence of IPTG was also restored by *ppiD *in multicopy (pPpiD), although the colonies grew up slower and remained smaller than those grown in the presence of IPTG. The growth-promoting effect of pPpiD was abolished by the introduction of a frameshift mutation that results in a premature stop at codon 173 of the plasmid-borne *ppiD *gene (pPpiDfs601). Thus, suppression of *surA skp *lethality elicited by pPpiD requires the intact *ppiD *gene. Multicopy *ppiD *also restored viability of *surA skp *cells in liquid media (Figure 2C). The *P_Llac-O1_*-*surA Δskp *strain ceased growth approximately 3.5 h after transfer into non-permissive media (LB without IPTG) but continued to grow when it carried pPpiD, although with slower rates during the mid- to late logarithmic phase. Western blot analysis indicated that PpiD is present in these cells at levels greater than that of chromosomally encoded PpiD (Figure 2D, lane 3 versus lanes 1 and 2). Thus, increased production of PpiD restores viability of *surA skp *cells but it does not completely compensate for the growth defect caused by the simultaneous lack of the SurA and Skp chaperones.

### Suppression of *surA skp *lethality does not require the parvulin domain but the membrane-localization of PpiD

The lethal phenotype of *surA skp *cells has been suggested to result from loss of periplasmic chaperone activity [10]. Consistent with this assumption, we found that the chaperone module of SurA (SurAN-Ct), which is devoid of any PPIase activity [2], is sufficient to fully complement the growth defect of the *P_Llac-O1_*-*surA Δskp *strain in the absence of IPTG (Figure 2B). To also dissect the activities and regions of PpiD required for complementation of *surA skp *lethality, we substituted amino acids G347 and I350 in its parvulin domain with alanine, generating the proteins PpiD^G347A ^and PpiD^I350A^, respectively. These mutations had originally been reported to effectively eliminate PPIase activity of PpiD and to result in the loss of its reported *surA *complementing function [18]. In addition, we completely deleted the parvulin domain from the protein, resulting in PpiDΔParv (Figure 2A). Only most recently, while this manuscript was in preparation, PpiD and its isolated parvulin domain have been shown to be devoid of PPIase activity [19]. However, because G347 and I350 are located at the peptide binding site of the parvulin domain, it was suggested that substrate binding to this domain is important for the *in vivo *function of PpiD.

**Figure 3:**
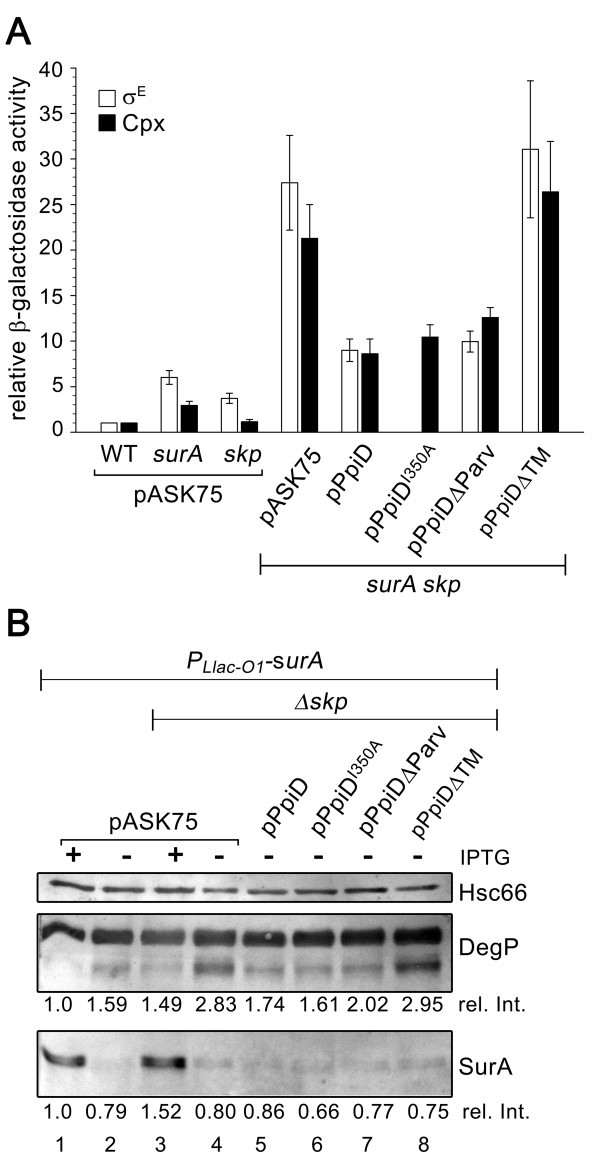
**Increased PpiD levels reduce σ^E ^and Cpx activity in *surA skp *cells**. (A) SurA-depletion strains carrying either the chromosomal σ^E^-dependent *rpoH*P3*::lacZ *or the Cpx-regulated *cpxP-lacZ *reporter fusions were cultivated at 37°C in LB buffered at pH 7.0 ± IPTG as described in *Methods*. Once growth of *P_Llac-O1_*-*surA Δskp *cells ceased in the absence of IPTG, samples were taken and assayed for σ^E ^and Cpx activities, respectively, by determining β-galactosidase activity. The strains contained either an empty vector (pASK75) or a plasmid encoding wild-type PpiD, PpiD^I350A^, PpiDΔParv, and PpiDΔTM (soluble His_6_-PpiD), respectively. The data shown are representative of at least two independent experiments. (B) Western blot detection of SurA and of DegP in crude extracts of cells after 240-minute growth at 37°C in LB ± IPTG. A volume of extracts equivalent to 4 × 10^7 ^cells was loaded onto each lane. Signal intensities were calculated using Hsc66 as the internal standard for each lane and are shown relative to those in the wild-type strain (rel. Int.). Western analysis was performed a minimum of two times for each σ^E ^and Cpx reporter assay, and data for one representative experiment are shown.

Both mutant proteins, PpiD^G347A ^and PpiD^I350A^, complemented the growth defect of *surA skp *cells just as well as wild-type PpiD, whereas PpiDΔParv complemented slightly less well in these assays (Figure 2B and 2C). Western blot analysis indicated however, that PpiDΔParv was present in the cells at significant lower levels than plasmid-encoded wild-type PpiD (Figure 2D, lane 5 versus lane 3), suggesting that the protein is less stable. We have confirmed that all three mutant PpiD proteins also restore growth of a *ppiD skp surA *triple mutant (additional file 2), demonstrating that the *surA skp *complementing activity does not depend on some residual function provided by chromosomally encoded wild-type PpiD. Together, these results show that the parvulin domain is not required for PpiD to function in rescuing *surA skp *cells from lethality. Unfortunately, we were unable to assess meaningfully if the N-terminal region of PpiD which shows sequence similarity to a substantial portion of the chaperone domain of SurA ([16-18] and additional file 1) contributes to this function, as a protein lacking the respective region (PpiDΔ69-201, Figure 3A) was present in the cells at even lower levels than PpiDΔParv (Figure 3D, lanes 7 and 8).

Finally, we asked if PpiD must be anchored to the inner membrane to function *in vivo*. Neither production of soluble N-terminally His_6_-tagged PpiD (PpiDΔTM) at a level similar to that of PpiDΔParv nor its production from pASKssPpiD at different inducer concentrations restored growth of *surA skp *cells (Figure 2, and data not shown). pASKssPpiD has also been used to produce and purify soluble His_6_-PpiD from the periplasmic fraction of *E. coli*, thus confirming the periplasmic location of the protein. As soluble His_6_-PpiD is functional *in vitro *(see below and [24]), these results suggest that the function of PpiD *in vivo *requires the protein to be anchored in the inner membrane.

### Overproduction of PpiD lowers folding stress in the cell envelope of *surA skp *cells

Previous studies suggested that the lethal phenotype of a *surA skp *mutant is caused by severe protein folding stress in the periplasmic compartment of the cells [10,25]. To determine whether increased PpiD levels restore viability of *surA skp *cells by counteracting folding stress in the cell envelope, we monitored the activities of the σ^E ^and Cpx stress pathways over time once growth of *P_Llac-O1_*-*surA Δskp *cells had leveled off in the absence of IPTG (time interval indicated in Figure 2C). At this time point, SurA was hardly detectable in the cells (Figure 3B), indicating that SurA had efficiently been depleted from the cells. During the course of the depletion of SurA in *Δskp *cells both the Cpx pathway and, as also reported previously [26], the σ^E^-dependent pathway were strongly induced (Figure 3A). The σ^E ^and Cpx activities were 4- to 6-fold increased in SurA-depleted *Δskp *cells (*surA skp *pASK75) relative to those of SurA-depleted wild-type cells (*surA *pASK75). This is also reflected in further increased levels of DegP (Figure 3B, lane 4 versus lane 2), whose gene is positively controlled by the σ^E ^and Cpx stress responses [27,28]. In *Δskp *cells that overproduced PpiD during the course of SurA depletion, σ^E ^and Cpx activities were significantly lower, being only 1.5- to 3-fold induced relative to the respective activities in *surA *cells. Consistent herewith, the level of DegP was lower in these cells than in *surA skp *cells that not overproduced PpiD but slightly higher than the DegP level in *surA *cells (Figure 3B, lane 5 versus lanes 4 and 2, respectively). Production of PpiDΔParv during the course of SurA depletion in *Δskp *cells reduced the σ^E ^and Cpx activities slightly less effectively and production of soluble His_6_-PpiD (PpiDΔTM), which does not rescue *surA skp *cells from lethality, further induced both stress responses (Figure 3A). Thus, only increased levels of membrane-anchored PpiD proteins dampen the strong response of the σ^E ^and the Cpx envelope stress signal transduction pathways to the simultaneous loss of SurA and Skp chaperone activity.

Taking advantage of the fact that overproduction of the outer membrane lipoprotein NlpE specifically induces the Cpx response [29] and that the Cpx pathway negatively controls expression of the σ^E ^regulatory components [30] we also asked whether down-regulation of σ^E ^activity alone would be sufficient to restore growth of *surA skp *cells. Indeed, the presence of multicopy *nlpE *during the course of SurA depletion in *Δskp *cells led to a further induction of the Cpx response and down-regulated σ^E ^activity to a similar extent as overproduction of PpiD (see additional files 3 and 4). Overexpression of *nlpE *even slightly improved cell growth in liquid media but it did not restore growth of *surA skp *cells on solid plates. Thus, Cpx-mediated repression of σ^E ^alone is not sufficient to restore *surA skp *cell viability.

### Effect of PpiD overproduction in *surA skp *cells on OMP biogenesis

**Figure 4:**
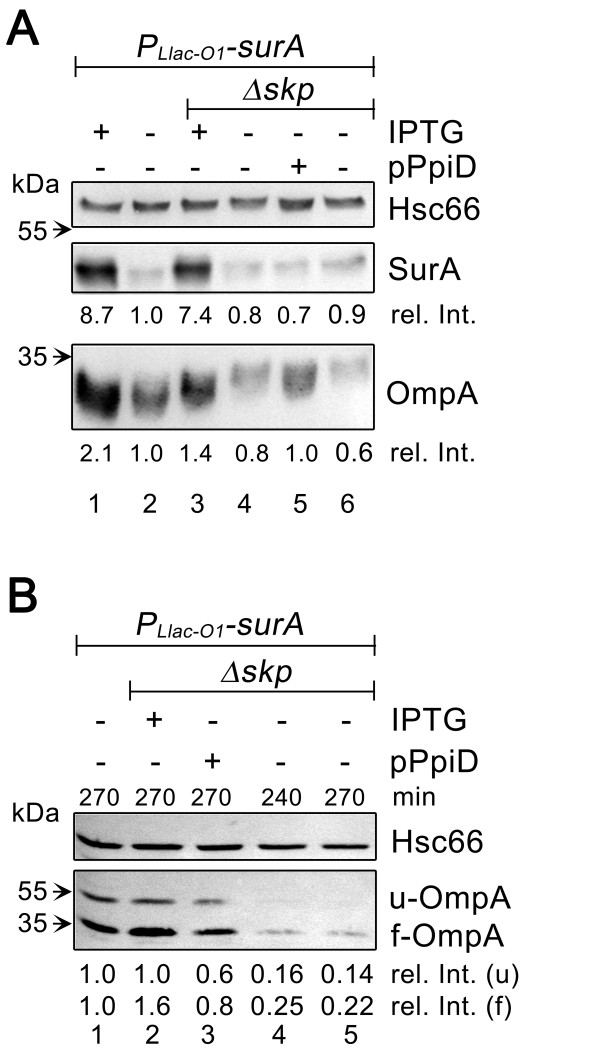
**Overproduction of PpiD in *surA skp *cells stimulates synthesis and folding of OmpA**. The SurA*-*depletion strains *P_Llac-O1_*-*surA *(SB44454) and *P_Llac-O1_*-*surA Δskp *(SB44452; *Δskp*) were grown at 37°C in LB buffered at pH 7.0 supplemented with 0.2% maltose ±of IPTG. Cells contained either pPpiD (+) or the empty vector pASK75 (-). The data shown are representative for a minimum of two independent experiments. (A) Total cellular levels of SurA and of OmpA in SurA-depletion strains grown for 240 min as described above. Extracts corresponding to 8 × 10^7 ^cells were loaded onto each lane and analyzed by western blotting. Signal intensities were calculated using cytoplasmic Hsc66 as the internal standard for each lane and are shown relative to those in the SurA-depleted *P_Llac-O1_*-*surA *strain (rel. Int.). (B) Levels of unfolded OmpA (u-OmpA) and folded OmpA (f-OmpA) species in SurA-depletion strains grown as described above. Culture samples corresponding to an equal number of cells were taken at the indicated time points and cell extracts prepared by gentle lysis. Samples of cell extracts corresponding to 1.3 × 10^8 ^cells were loaded onto each lane and analyzed by western blotting. Relative signal intensities (rel. Int.) for u-OmpA (u) and f-OmpA (f) were calculated as in A.

The reduction of σ^E ^activity in *surA skp *cells elicited by higher levels of PpiD suggests that PpiD in these cells directly or indirectly affects OMP biogenesis. σ^E ^positively controls the production of small non-coding RNAs, which down-regulate OMP synthesis by translational repression [31], and decreased levels of OMPs in SurA-deficient cells therefore reflect defects in both OMP synthesis and assembly [6]. We asked if conversely, the decrease in σ^E ^activity in PpiD overproducing *surA skp *cells correlated with increased levels of the major OMP OmpA. Western blot analysis of crude cell extracts confirmed a slight increase in the level of OmpA in these cells as compared to *surA skp *cells (Figure 4A lane 5 versus lanes 4 and 6, respectively), suggesting that in the absence of SurA and Skp increased levels of PpiD stimulate OmpA synthesis and/or stability. To substantiate this result and to explore a possible influence of PpiD on OmpA folding in *surA skp *cells, we examined the consequence of PpiD overproduction on the OmpA folding state during the course of SurA depletion in *Δskp *cells. The OmpA folding state can be conveniently followed by a shift in the apparent mass on SDS polyacrylamide gels. The folded β-barrel domain of OmpA is stable in 2% SDS and migrates faster than unfolded OmpA if not heat-denatured prior to electrophoresis [32]. OMPs were prepared by gentle lysis to preserve their native conformation [33] and OmpA folding intermediates were detected by western blotting (Figure 4B). In contrast to previous work showing that unfolded OmpA accumulates in *surA skp *double null cells [26], we found the conditional *surA skp *mutant to contain significantly reduced levels of both, folded and unfolded forms of OmpA (lanes 4 and 5). This difference may reflect the use of a different SurA depletion strategy or the presence of higher levels of DegP protease activity in the strain used here, or both. In any case, the amount of folded OmpA was clearly increased in *surA skp *cells that overproduced PpiD (lane 3) and was almost as high as that in *surA *cells (lane 1). Thus, in *surA skp *cells both synthesis and folding of OmpA is stimulated by increased PpiD levels. Together, these results suggest that in the absence of both chaperones, SurA and Skp, overproduction of PpiD can, at least in part, counteract the defects in the biogenesis of OmpA and possibly of other OMPs.

### PpiD has *in vitro *chaperone activity

**Figure 5:**
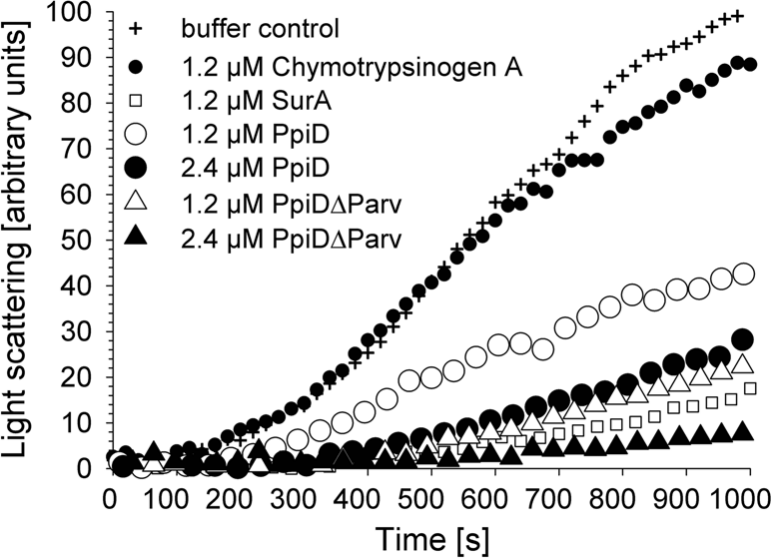
**The PpiD and PpiDΔParv proteins exhibit chaperone activity *in vitro***. Thermal aggregation of citrate synthase (0.15 μM monomer) at 43°C in the presence of SurA (positive control), Chymotrypsinogen A (negative control), and the soluble PpiD and PpiDΔParv proteins was observed by light scattering at 500 nm.

The above findings suggest that suppression of the lethal *surA skp *phenotype by overproduction of PpiD does not simply result from regulatory events in response to increased PpiD levels but rather from functional complementation of the *surA skp *caused deficiency. As the defects of the *surA skp *double mutant are thought to result from lack of periplasmic chaperone activity [10], we asked whether the PpiD and PpiDΔParv proteins provide such an activity by examining their capability to prevent aggregation of thermally denatured citrate synthase, a classic *in vitro *assay for chaperone function [34]. SurA had previously been shown to possesses this activity [2] and was used as a control. When citrate synthase was thermally denatured in the presence of an 8-fold molar excess of SurA (based on citrate synthase monomer) aggregation was significantly reduced (Figure 5). Chymotrypsinogen A, which served as a negative control, showed no or only minor effects at this concentration. In contrast, an 8-fold excess of PpiD reduced aggregation of citrate synthase significantly, although less effectively than SurA, requiring 2-fold higher concentrations to have roughly the same effect. PpiDΔParv finally, which lacks the PPIase domain (Figure 2A), protected citrate synthase about 2-fold more effectively from aggregation than intact PpiD, being almost as effective as SurA. The observed chaperone effects are not caused by protease contamination, as the same amount of citrate synthase was present in samples taken throughout a 1200 s incubation period with a 16-fold excess of PpiD proteins (as judged by SDS-PAGE and silver stain; data not shown). Thus, PpiD exhibits a chaperone activity that is carried in the non-PPIase regions of the protein. The finding that PpiDΔParv complements the growth defect of a *surA skp *mutant less well than full-length PpiD (Figure 2C) although it exhibits stronger *in vitro *chaperone activity (Figure 5) likely relates to the presence of lower levels of PpiDΔParv than of plasmid-encoded intact PpiD in these cells (Figure 2D). The overall chaperone activity provided by PpiDΔParv in the cells may thus be weaker than that provided by the overproduced intact PpiD.

### PpiDΔParv complements the growth defect of an *fkpA ppiD surA *triple mutant

**Figure 6:**
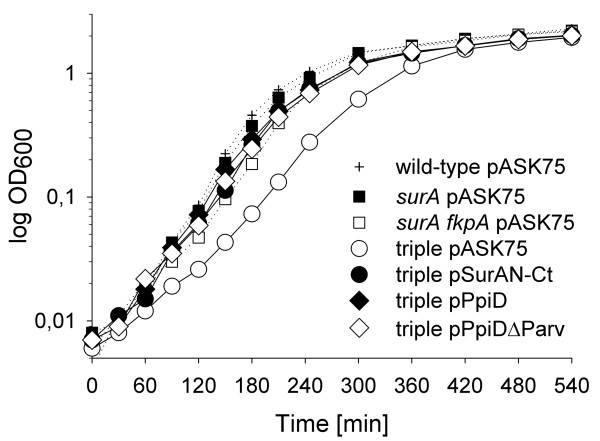
**Growth complementation of an *fkpA ppiD surA *triple mutant**. Growth of the *fkpA ppiD surA *(SB11116; triple), *fkpA surA *(SB11114), and *surA *(CAG24029) PPIase mutants and of wild-type (CAG16037) in LB at 37°C was assayed by monitoring the OD_600 _during shaking culture.

To provide further *in vivo *evidence for the existence of a chaperone activity of PpiD we took advantage of a phenotype that has previously been shown to be associated with inactivation of *ppiD*. Such a phenotype is exhibited by an *fkpA ppiD surA *triple mutant, which displays growth defects during mid- to late exponential phase in liquid culture, while all double mutant combinations including these genes grow normally [20]. The *fkpA *gene codes for the periplasmic folding factor FkpA, which like SurA exhibits PPIase and chaperone activity [35,36]. Our complementation analysis showed that both the SurAN-Ct protein, which only exhibits chaperone activity [2], and PpiDΔParv restore growth of the *fkpA ppiD surA *mutant as well as intact PpiD (Figure 6). This demonstrates that the growth phenotype of the triple PPIase mutant is not due to loss of PPIase activity but to loss of chaperone function. It also shows that PpiD shares this function with SurA and FkpA. As in SurA, the chaperone activity is carried solely in the non-parvulin regions of the protein (PpiDΔParv).

### Lack of PpiD confers increased temperature-sensitivity in a *degP *mutant

**Figure 7:**
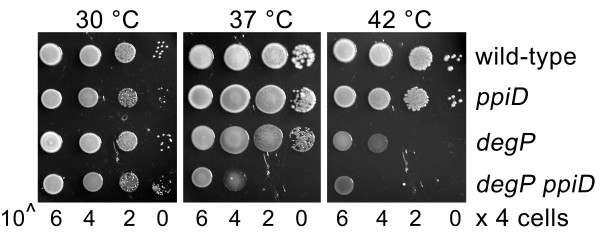
**Inactivation of *ppiD *confers increased temperature sensitivity in a *degP *mutant**. Growth analysis of wild-type (CAG16037), *degP::kan *(SB44964), *ppiD::*Tn*10 *(SB44741), and *degP::kan ppiD::*Tn*10 *(SB44970) cells. Cells were grown overnight at 30°C and after dilution spotted on LB plates. Plates were incubated overnight at the indicated temperature.

The periplasmic protease DegP also acts as a chaperone [15,37] and the simultaneous lack of DegP and SurA gives a synthetically lethal phenotype [10]. We therefore asked whether similarly a chaperone function of PpiD may be disclosed by the combined deletion of *ppiD *and *degP*. DegP-deficient strains display a temperature-sensitive phenotype at temperatures above 37°C [38]. Accordingly, we compared the growth of *degP *and *ppiD *single mutants with that of a *degP ppiD *double mutant at 30, 37, and 42°C (Figure 7). As expected, lack of DegP compromised cell growth above 37°C. In contrast, the *ppiD *single mutant showed wild-type growth at all temperatures. However, the *degP ppiD *double mutant was more temperature sensitive than the *degP *single mutant and grew normally only at 30°C. Thus, *degP ppiD *mutants show a synthetic conditional phenotype at temperatures greater than 30°C.

## Discussion

### PpiD is a SurA-like multidomain chaperone

To date, four representatives of the three major families of PPIases are known to exist in the periplasm of *E. coli*: the cyclophilin PpiA [39], the FKBP-like protein FkpA [35], and the parvulin-like proteins PpiD [18] and SurA [6-8]. In addition to PPIase activity, SurA and FkpA also exhibit prolyl isomerase-independent chaperone activity [2,36] and the major function of SurA in the maturation of the integral β-barrel OMPs actually is that of a chaperone [2]. While PpiD has also been implicated in OMP biogenesis, the biochemical activity required for this function was reported to be a PPIase activity carried in its parvulin domain [40]. A chaperone activity has so far not been demonstrated for either PpiD or PpiA.

In this study we for the first time directly demonstrate, both *in vitro *and *in vivo*, that PpiD exhibits a PPIase-independent chaperone activity that resides in the N- and/or C-terminal regions of the protein. The parvulin domain of PpiD is neither required for function *in vivo *nor for chaperone activity *in vitro*, as a PpiD protein lacking this domain fully complements the growth defect of an *fkpA ppiD surA *triple mutant and protects citrate synthase from thermal aggregation even more effectively than wild-type PpiD. In addition, these results show that a catalytic prolyl isomerase activity plays no major role for the function of PpiD *in vivo*. This conflicts with previous results [40] but is consistent with most recent data showing that the parvulin domain of PpiD is devoid of detectable PPIase activity *in vitro *[19].

The chaperone function of PpiD is most likely carried in its N-terminal region, which shares sequence similarity with the N-terminal region of SurA (see additional file 1A; [16-18]) and thus with a substantial part of the SurA chaperone module [2]. Model structures of this region of PpiD generated by alignment based as well as by automated three-dimensional homology modeling (see additional file 1, C and D) show some deviation from the crystal structure of the SurA chaperone module mainly in the helix 1-helix 2 and the helix 3-helix 4 interconnecting loop regions. Interestingly, whereas the chaperone module of SurA lacks sequence similarity with the structurally homologous chaperone domain of the *E. coli *cytosolic Trigger factor (TF) [41], the predicted helix 1-loop-helix 2 region of PpiD shows similarity on the amino acid level with the corresponding region of TF (24.1% identity between regions 43-121 and 295-371 of PpiD and TF, respectively; see additional file 1, B and E).

The similarities in sequence and predicted structure between PpiD, SurA and TF suggest that PpiD contains a conserved SurA-like chaperone module. However, for a complete chaperone active module the region of PpiD that would correspond to the C-terminal helix of SurA still needs to be identified. As an integral element of the conserved module structure this helix is indispensable for the stability and activity of SurA [2,42] and presumably also of other members of this family of chaperones. The C-terminal helix of SurA was originally identified as the stabilizing region of the protein as it is very basic (predicted isoelectric points of 10.5) as compared to the rather acidic N-terminal region (predicted isoelectric point 5.3) [2]. Similarly, the corresponding helix in the chaperone domain of TF is rather basic as opposed to the rest of the module (predicted isoelectric points of 8.4 and 4.7, respectively). Finally, the N-terminal region of PpiD is acidic too (predicted isoelectric point of 4.7) and therefore the single basic region of the protein which is located in the C-terminal domain (amino acids 511-560, predicted isoelectric point of 10) and is predicted to be rich in α-helical secondary structure, would be a primary candidate for the stabilizing region. Taken together, all indications are that PpiD is a membrane-anchored SurA-like multidomain chaperone, which like SurA combines a conserved chaperone module with an inactive parvulin domain. Different from SurA however, PpiD lacks a second active parvulin domain and instead contains a C-terminal domain, whose function remains to be determined.

### Role of PpiD in the periplasm

PpiD was previously reported to be redundant in function with SurA in the maturation of OMPs [18]. Our results however, establish that PpiD plays no major role in the biogenesis of OMPs and that it cannot compensate for lack of SurA in the periplasm. In addition, PpiD differs from SurA in that it requires to be anchored in the inner membrane to function *in vivo *whereas SurA is functional both in a soluble and in a membrane-anchored state (S. Behrens-Kneip, unpublished results). Then again, *ppiD *in multicopy suppresses the *surA skp *caused deficiencies. The strong induction of the σ^E ^and Cpx stress pathways during the course of depletion of SurA from Δ*skp *cells is significantly reduced by simultaneous overproduction of PpiD. This suggests that increased levels of PpiD rescue *surA skp *cells from lethality by counteracting the severe folding stress in the cell envelope which results from the loss of periplasmic chaperone activity. We cannot formally eliminate the possibility that the observed effects result from a response of (an)other known or still to be identified regulatory pathway(s) to increased levels of PpiD in the inner membrane. Such regulatory mechanisms may, for instance, induce periplasmic protease activity that reduces folding stress by protein degradation. However, they would not readily explain our observation that PpiD overproducing *surA skp *cells contain higher levels of folded forms of OmpA even though they lack two of three chaperones critical for OMP folding. The third OMP chaperone, DegP, appears to interact preferentially with OMPs that already contain substantial levels of folded structure [15] and would thus be expected to predominantly assist in late steps of OMP folding. Moreover, since DegP levels in *surA skp *cells are reduced by overproduction of PpiD it seems implausible that DegP is responsible for the observed effect on OmpA folding. This, together with our finding that PpiD has chaperone activity *in vitro *leads us to suggest that PpiD, when present at sufficient levels, is able to partially compensate for the simultaneous loss of SurA and Skp chaperone function.

But why would PpiD promote the folding of OmpA in a *surA skp *double mutant but have no discernable impact on OMP folding in the respective *surA *and *skp *single mutants? We believe that this effect is due to overlapping substrate specificities but yet distinct roles of these chaperones in the periplasm, as has also been suggested for the SurA and Skp chaperones [5,26]. Both SurA and Skp interact with unfolded major OMPs [2,43] and facilitate their biogenesis, yet they cannot functionally substitute one another in the cell (Figure 1 and our unpublished data) and are thought to act in parallel pathways of OMP folding [5,26]. The peptide binding specificity of PpiD has been shown to overlap with that of SurA but to be less specific [44], suggesting that PpiD is capable of interacting with a broader range of substrates. Thus, while unfolded major OMPs obviously are no preferred substrates of PpiD, they may still effectively interact with PpiD for folding in the absence of the competing chaperones SurA and Skp. In this context it is important to mention, that overproduction of PpiD does not restore viability of a *surA degP *double mutant (S. Behrens-Kneip, unpublished results). This suggests that, when overproduced in *surA skp *cells, PpiD compensates for the lack of Skp upstream of DegP in the proposed Skp/DegP branch of protein folding rather than for the lack of SurA. The magnitude of suppression of the *surA skp *phenotypes elicited by multicopy *ppiD *and the additive phenotypes of the *ppiD degP *and *skp ppiD *double mutants described in this work are in support of this notion. Moreover, direct evidence for partially overlapping functions of the membrane-anchored PpiD and the soluble Skp comes from the recent finding that both proteins interact with OMP polypeptides very early during translocation and promote their release from the SecYEG translocation channel [12,14,24].

It has recently been proposed that PpiD is a periplasmic gatekeeper of the Sec translocon responsible for newly translocated OMPs [24]. Our work agrees with and refines this assumption, as it shows that PpiD exhibits the requisite chaperone activity for such a function, that this function is not preferentially directed at folding of OMPs, and that PpiD cooperates with SurA, Skp, FkpA and DegP in mediating protein folding in the periplasmic compartment of the cell. We suggest that the role of PpiD is to assist in the initial periplasmic folding events of many newly secreted envelope proteins.

In the cytosol, the folding of newly synthesized proteins is initiated by the ribosome-associated chaperone TF [45,46]. Of note, PpiD and TF show some interesting analogies. First, similar to PpiD TF is composed of three domains: an N-terminal ribosome-binding domain, a central FKBP-like PPIase domain, and a C-terminal chaperone module which is structurally homologous to the chaperone module of SurA [41,47] and, as outlined above, shows sequence similarity with the N-terminal putative chaperone region of PpiD. Second, TF associates with the ribosome to sequester and protect polypeptides just as they emerge from the peptide exit tunnel [46] and this association is crucial for its *in vivo *function [48]. PpiD on the other hand, is anchored in the inner membrane and interacts with newly translocated polypeptides that emerge from the periplasmic exit site of the Sec translocon [24] and according to our data, the anchoring of PpiD in the membrane is required for its function *in vivo*. Third, TF is dispensable for cell viability and a deletion of the *tig *gene confers a discernable phenotype only in combination with a mutation of the *dnaK *gene for the cytosolic chaperone DnaK [45]. Likewise, lack of PpiD gives a discernable phenotype only in cells with already compromised periplasmic chaperone activity, such as in the *fkpA ppiD surA *triple mutant and in the *degP ppiD *and *ppiD skp *double mutants. Finally, the amino acid sequence pattern of known PpiD binding peptides [44] resembles that of the peptide binding motifs identified for the cytosolic chaperones TF and DnaK, consisting of a central patch of hydrophobic amino acids flanked by positively charged amino acids [49]. Altogether, we speculate that PpiD may represent the periplasmic counterpart of TF. Its fixed localization in the inner membrane not necessarily conflicts with such a function, as it may provide a local enrichment of the binding partners but still allows PpiD to dynamically interact with and cycle on and off its interaction partners by lateral diffusion in the membrane, just as it is the hallmark of TF function on translating ribosomes [50]. Further detailed studies on the interaction of PpiD with both the Sec translocation machinery and translocating polypeptides as well as the analysis of the structure-function relationship in PpiD are needed to ascertain its real function in the periplasm.

## Conclusions

This study for the first time directly demonstrates that PpiD functions as a chaperone and that its previous classification as a folding factor for OMPs must be revised. PpiD appears to belong to the SurA-like family of chaperones but different from SurA it plays no major role in the maturation of OMPs. A biochemical capability of PpiD to also assist the folding of OMPs becomes relevant only in the absence of both chaperones for unfolded OMPs, SurA and Skp. In addition, the role of PpiD in the periplasm appears to be restricted to folding events that take place in close proximity to the inner membrane, as only membrane-anchored PpiD functions *in vivo*. Taken together, our data are in line with the recently proposed role of PpiD as a periplasmic gatekeeper of the Sec translocon [24], as they suggest that it acts as a chaperone for initial folding events of many newly exported proteins. We speculate that PpiD may have a role at the periplasmic exit site of the Sec translocon similar to that of TF at the exit site of the translating ribosome.

## Methods

### Media and growth conditions

Luria-Bertani (LB) media were prepared as described [51]. Ampicillin (Ap), chloramphenicol (Cm), kanamycin (Kan), spectinomycin (Spec), and tetracycline (Tc) were used at final concentrations of 100, 20, 30, 50 and 10 μg ml^-1^, respectively. For assaying β-galactosidase activity in *cpxP*-*lacZ *reporter strains the medium was buffered with 100 mM sodium phosphate to a pH of 7.0, at which *cpxP *transcription, which is affected by extracellular alkaline pH, is induced to a medium level [57]. Strains were grown at 37°C with aeration unless noted otherwise.

### Strains

Strains used in this work are listed in Table 2. Mutant alleles were moved into the appropriate strains either by general transduction using phage *T4-GT7 *[52] or by P1 transduction [53]. The presence of the mutant alleles in recombinants was verified by PCR. To generate SurA-depletion strains the chromosomal *surA *gene was placed under the control of the IPTG-inducible promoter *P_Llac-O1 _*[23] by gene replacement as described previously [54]. A ~3.1 kb DNA fragment bearing an *Ω:: spectinomycin-P_Llac-O1 _*fusion flanked by approximately 500 bp of *imp *and *surA *sequence, respectively, was obtained from pΩSurA by cleavage with *Eco*RI and partial digest with *Hin*dIII. *E. coli *KM22 was electroporated with the purified fragment. Recombinants were selected on LB/Spec and used as donors for transduction of the *Ω::spec*-*P_Llac-O1_*-*surA *locus into the appropriate strains. The final *Ω::spec*-*P_Llac-O1_*-*surA *strains were transformed with pPLT13 to provide the LacI repressor protein.

**Table 2:** Strains used in this study

Strain	Genotype	Source, reference, donor strain
CAG16037	MC1061 ϕλ[*rpoH P3::lacZ*]	[56]
CAG24029	CAG16037 *surA::*Tn*10d*Cm	[6]
CAG33398	MC1061 λRS88(*cpxP-lacZ*)	C.A. Gross laboratory
CAG37057	CAG16037 *Δskp zae-502::*Tn*10*	C.A. Gross laboratory
CAG44102	MC4100 *surA::*Tn*10d*Cm *slyD1 zhg::*Tn*10*; Cm^R^, Tc^R^	[2]
KM22	*argE3 his-4 leuB6 thr-1 ara-14 galK2 lacY1 mtl-1 xyl-5 thi-1 rpsL31 tsx-33 supE44 Δ(recC ptr recB recD)::*P*_lac_-bet exo kan*	[54]
SB10042	CAG24029 *ppiD::*Tn*10*	This study; donor MC4100 *ppiD::*Tn*10 *(T. Silhavy)
SB11019	CAG33398 *Ω::spec*-*P_Llac-O1_*-*surA *pPLT13	This study
SB11067	CAG33398 *ppiD::*Tn*10*	This study; donor MC4100 *ppiD::*Tn*10 *(T. Silhavy)
SB11069	CAG33398 *surA::*Tn*10d*Cm	This study; donor CAG24029
SB11072	SB44080 *ppiD::kan*	This study; donor JW0431 [59]
SB11075	SB11069 *ppiD::*Tn*10*	This study; donor MC4100 *ppiD::*Tn*10 *(T. Silhavy)
SB11114	CAG24029 *fkpA::kan*	This study; donor JW3309 [59]
SB11116	SB10042 *fkpA::kan*	This study; donor JW3309 [59]
SB11179	CAG33398 *ppiD::kan*	This study; donor JW0431 [59]
SB44080	CAG33398 *Δskp zae-502::*Tn*10*	This study; donor CAG37057
SB44451	CAG37057 *Ω::spec*-*P_Llac-O1_*-*surA*	This study
SB44452	CAG37057 *Ω::spec*-*P_Llac-O1_*-*surA *pPLT13	This study
SB44454	CAG16037 *Ω::spec*-*P_Llac-O1_*-*surA *pPLT13	This study
SB44741	CAG16037 *ppiD::*Tn*10*	This study; donor MC4100 *ppiD::*Tn*10 *(T. Silhavy)
SB44913	CAG16037 *ppiD::kan*	This study; donor JW0431 [59]
SB44914	CAG37057 *ppiD::kan*	This study; donor JW0431 [59]
SB44961	SB44451 *ppiD::kan *pACLacI	This study; donor JW0431 [59]
SB44964	CAG16037 *degP::kan*	This study; donor JW0157 [59]
SB44970	SB44741 *degP::kan*	This study; donor JW0157 [59]
SB44997	CAG44080 *Ω::spec*-*P_Llac-O1_*-*surA *pPLT13	This study

### Plasmids

Plasmids used in this study are listed in Table 3. To make pΩSurA, the sequences flanking the *Ω::spec*-*P_Llac-O1 _*cassette in plasmid pBA106 [55] were replaced by portions of the *imp-surA *locus corresponding to nucleotides -581 to -35 (*imp3'*, 497 bp) and nucleotides -26 to 508 (*surAN*, 534 bp), respectively, relative to the *surA *translational start codon. Fragment *imp3' *was amplified by PCR from purified MC1061 genomic DNA using the primers 5'-GGATTGCGTGGCGGAATTCAGTACG-3' and 5'-ACCGCACTGCGGATCCCGTGGTAAATC-3'. The *Eco*RI/*Bam*HI-cleaved product was ligated into the corresponding sites of pBA106. Subsequently, the *surAN *fragment was obtained from pSurAN [2] by *Nco*I/*Hin*dIII cleavage and cloned into the corresponding sites downstream of *Ω::spec*-*P_Llac-O1 _*in the above intermediate. pASKSurAN-Ct was constructed by cloning a *Pst*I/*Bgl*II fragment of pSurAN-Ct [2] into the corresponding sites of pASKSurA [2]. To yield pPpiD, the *ppiD *gene and its promoter region was PCR amplified from the MC1061 chromosome using the primers 5'-GTGCTGCCCATATGGGCCGCAACCCG-3'and 5'-TTTTGCGAGGAAGCTTCAGGA TTATTGC-3'. The PCR fragment was cleaved with *Nde*I/*Hin*dIII and cloned into the *Nde*I and *Hin*dIII sites of pTrc99a, thereby removing the plasmid encoded *lacI^q ^*gene and *P_trc _*promoter sequences. Plasmids pPpiD^G347A ^and pPpiD^I350A ^were created by replacing the codons 347 and 350 of *ppiD *to codons for alanine by QuikChange site directed mutagenesis (Stratagene, La Jolla, CA) using the primer pair 5'-CAAATCTTCGGTCGCTTTCCTG-3'/5'-CAGGAAAGCGACCGAAGATTTG-3' and 5'-CGGTTTCCTGGCTGTACGTCTGG-3'/5'-CCAGACGTACAGCCAGGAAACC-3', respectively. Plasmid pPpiDΔ69-201 was made by deletion of a *Pvu*I/*Hpa*I fragment from pPpiD. pPpiDΔParv was constructed as follows: a second *Eco*RV site was introduced at nucleotides 1062-1068 of *ppiD *by QuikChange mutagenesis of pPpiD using primers 5'-GTCTGGACGATATCCAGCCAGCGAAAG-3' and 5'-CTTTCGCTGGCTGGATATCGTCCAGAC-3'. In the resulting plasmid, the parvulin domain encoding sequence of *ppiD *was flanked by *Eco*RV sites. Deletion of the *Eco*RV fragment resulted in pPpiDΔParv. Plasmid pPpiDfs601 was made by cleavage of pPpiD with *Kpn*I, removal of the resulting 3'-overhangs with DNA polymerase I Klenow fragment, and subsequent ligation. Plasmid pASKssPpiD for the production of a soluble periplasmic N-terminally hexa-His-tagged PpiD protein was constructed in three steps. First, a *Bam*HI site was introduced at codons 33-34 of *ppiD *by QuikChange mutagenesis of pPpiD using primers 5'-GCGTGAGTGGATCCCTGATTGGCGGA-3' and 5'-TCCGCCAATCAGGGATCCACTCACGC-3'. Second, the *Bam*HI/*Hin*dIII fragment of the resulting plasmid, encoding PpiD without the transmembrane segment, was cloned into the *Bam*HI/*Hin*dIII sites of a pASKSurA plasmid that carried a *Sac*I site at codons 22-23 of *surA *[2]. Third, the 5'-phosphorylated oligonucleotides 5'-CCATCACCATCACCATCACG-3' and 5'-GATCCGTGATGGTGATGGTGATGGAGCT-3' were annealed and cloned into *Sac*I/*Bam*HI of the above intermediate, thereby placing a hexa-His sequence between the signal peptide sequence of *surA *and codons 34 to 623 of *ppiD*. To make pASKssPpiDΔParv, the *Sph*I/*Pst*I fragment of pASKssPpiD bearing the parvulin domain encoding sequence was replaced by a *Sph*I/*Pst*I fragment derived from plasmid pPpiDΔParv. To make pPpiDΔTM, a 1350 bp-fragment carrying the *surA signal sequence-his_6_-ppiD *fusion was PCR amplified from pASKssPpiD using primers 5'-CATTGATAGAGTTACGTAACCACTCCC-3' and 5'-CACTTTCTGCTGCAGCGCG-3'. The product was cleaved with *Sna*BI/*Pst*I and cloned into the *Stu*I and *Pst*I sites of pPpiD. To create plasmid pSkp, a 1722 bp *Xho*I/*Nde*I fragment derived from plasmid pMP1 was cloned into the corresponding sites of pQE60 thereby removing the plasmid-encoded *P_T5_/O_lac _*promoter/operator sequences. All plasmid sequences were confirmed by DNA sequencing.

**Table 3:** Plasmids used in this study

Plasmid	Genotype	Source, reference
pACLacI	pACYC184 derivative with *lacI^q^*; Cm^R^	This study
pASK75	vector, P/O*_tet_*, *tetR*, ColEI ori; Ap^R^	[60]
pASKSurA^a^	*surA *gene in pASK75; Ap^R^	[2]
pASKSurAN-Ct^b^	*surAN-Ct *fusion from pSurAN-Ct [2] in pASK75; Ap^R^	This study
pASKssPpiD	*surA signal sequence*-his_6_-*ppiD *(codons 34-623) fusion in pASK75; Ap^R^	This study
pASKssPpiDΔParv	pASKssPpiDΔ252-355; Ap^R^	This study
pΩSurA	*Ω::spec-P_Llac-O1 _surA *in pUC18; Ap^R^; Spec^R^	This study
pMP1	*skp *gene region of *E. coli *MC1061 (corresponding to nucleotides 199495-201937 of the *E. coli *MG1655 genome^c^) in pSU18; Cm^R^	Gross laboratory
pPLT13	mini-F carrying *lacI^q^*; Kan^R^	[61]
pPpiD	*ppiD *gene and promoter of *E. coli *MC1061 (corresponding to nucleotides 460852-463020 of the *E. coli *MG1655 genome^c^) in pTrc99A (*lacI^q ^*and *P_trc _*sequences deleted); Ap^R^	This study
pPpiD^I350A^	pPpiD carrying mutation I350 to A; Ap^R^	This study
pPpiD^G347A^	pPpiD carrying mutation G347 to A; Ap^R^	This study
pPpiDΔ69-201	pPpiDΔ69-201; Ap^R^	This study
pPpiDΔParv	pPpiDΔ252-355; Ap^R^	This study
pPpiD(ΔTM)	*surA signal sequence*-his_6_-*ppiD *(codons 34-623) fusion in pPpiD; Ap^R^	This study
pPpiDfs601	pPpiD with a frameshift mutation in *ppiD *that generates a stop at codon 173; Ap^R^	This study
pQE60	C-His_6 _fusion vector, *P_T5_/O_lac_*, ColEI ori; Ap^R^	Qiagen
pSkp	*skp *gene and *skp *promoter of *E. coli *MC1061 (corresponding to nucleotides 200073-201801 of the *E. coli *MG1655 genome^c^) in pQE60 (*P*_T5/_O_lac _deleted); Ap^R^	This study
pTrc99a	Expression vector, *P_trc_*, ColEI ori; Ap^R^	Amersham Pharmacia

^a,b^Referred to as pSurA and pSurAN-Ct, respectively, in the text.

^c^accession number NC_000913 [62]

### Assay of susceptibility to SDS/EDTA

The sensitivity of the strains to SDS/EDTA was determined in plating assays as previously described [2]. The efficiency of plating was calculated from the colony count after incubation at 37°C for 24-48 h. A minimum of three experiments were performed for each strain and condition.

### Spot dilution assays

SurA-depletion strains were freshly transformed with the required plasmids and were grown overnight at 37°C in selective LB containing 1 mM IPTG. Overnight cultures were adjusted to an optical density at 600 nm (OD_600_) of 4.0 and 10-fold serially diluted with IPTG-free LB. Ten microlitres of the 10^-1^, 10^-3^, 10^-5^, and 10^-7 ^dilutions were spotted on LB ± 1 mM IPTG plates supplemented with the appropriate antibiotics and incubated at 37°C for 16-24 h. To test for temperature sensitivity, strains were grown overnight at 30°C in LB and were diluted and spotted on LB plates as described above.

### SurA depletion *in vivo*

SB44452 or SB44997 were freshly transformed with the appropriate plasmids and grown overnight at 37°C in LB/Ap/Kan/Spec (buffered at a pH of 7.0, if required) supplemented with 1 mM IPTG and 0.2% (w/v) maltose to induce expression of the maltoporin LamB. Two milliliters of each overnight culture were pelleted in a microcentrifuge and were washed three times in 2 ml of LB to remove IPTG from the cells. The washed cultures were then diluted to an OD_600 _of 0.01 into 50 ml of LB/Ap/Kan ± 1 mM IPTG. These pre-cultures were grown for 4-5 cell generations with shaking in a gyratory water bath at 37°C and diluted into fresh LB/Ap/Kan ± 1 mM IPTG to an OD_600 _of 0.005. Aliquots were sampled for β-galactosidase assays, for western blot analysis, and for the preparation of OmpA folding intermediates at the indicated time points after the second sub-culturing and processed as described below.

### β-galactosidase *assays*

Eσ^E ^and Cpx pathway activities were assayed by monitoring the β-galactosidase activity resulting from the expression of the chromosomal σ^E^- and Cpx-dependent reporter fusions *rpoH*P3*::lacZ *[56] and *cpxP-lacZ *[57], respectively, in cells growing in LB medium (buffered at pH 7.0 for Cpx assays) at 37°C. Overnight cultures were diluted to an OD_600 _of 0.005 into fresh media and grown with shaking in a gyratory water bath at 37°C. Duplicate samples (0.5 ml) were taken throughout the early exponential phase of the growth curve (OD_600 _= 0.08-0.4) and β-galactosidase activity was measured by the standard assay [53]. Eσ^E ^and Cpx activities shown in Figure 1 were determined from the slope on the line of a differential plot of β-galactosidase activity in 0.5 ml of culture versus OD_600 _and normalized to the wild-type case. In Figure 3, the average β-galactosidase activity/OD_600 _(Miller Units) was calculated and normalized to that of wild-type. Statistical analysis was performed using a Student's *t*-test.

### Western blot analysis

Whole cell extracts were prepared by resuspending cells in urea protein sample buffer (8 M urea, 200 mM Tris-Base, 200 mM DTT, 2% SDS, 0.02% bromphenol blue) followed by short sonication and heating of the sample to 95°C for 10 min. Extracts from equal numbers of cells were run on SDS-polyacrylamide gels and transferred to nitrocellulose membranes. The membranes were probed with dilutions of rabbit polyclonal antisera raised against SurA (1:10 000), PpiD (1:10 000), DegP (1:20 000), Hsc66 (1:20 000), LamB (1:3000), and with mouse monoclonal antibodies raised against OmpA (1:500), respectively. Alkaline phosphatase conjugated goat anti-rabbit and anti-mouse IgGs (Sigma, 1.10 000 dilutions), respectively, served as secondary antibodies. They were visualized by incubating the blots in reaction buffer (100 mM Tris-HCl, pH 8.8, 100 mM NaCl, 5 mM MgCl_2_, 37.5 μg/ml nitro blue tetrazolium, 150 μg/ml 5-bromo-4-chloro-3-indolyl phosphate). Signal intensities were quantified using ImageJ software http://rsb.info.nih.gov/ij/. Hsc66 and MalE were used as the internal standard for each lane. Experiments were repeated a minimum of two times for each strain and condition, and data for one representative experiment are shown.

### Preparation of OmpA folding intermediates

During the course of SurA depletion, samples corresponding to an equal number of cells were harvested by centrifugation and immediately frozen in a dry ice/ethanol bath. Folded and unfolded OmpA folding intermediates were isolated by gentle lysis as previously described [33]. Samples were mixed with protein sample buffer (3% SDS, 10% glycerol, 5% β-mercaptoethanol in 70 mM Tris, HCl, pH 6.8), heated to 37°C for 10 min and loaded onto 12.5% SDS-polyacrylamide gels. Electrophoresis was performed at 50 V and OmpA intermediates were detected by Western blot analysis as described above.

### Protein purification

N-terminally His_6_-tagged PpiD proteins and C-terminally His_6_-tagged SurA were produced in *E. coli *CAG44102 from pASKssPpiD, pASKssPpiDΔParv and pASKSurA, respectively, and purified from the periplasmic fraction by affinity chromatography on Ni^2+^-chelating sepharose as previously described [2]. Protein concentrations were determined by UV absorbance at 280 nm using absorption coefficients of 36 270 M^-1 ^cm^-1^, 26 740 M^-1 ^cm^-1 ^and 29 450 M^-1 ^cm^-1 ^for His_6_-PpiD, His_6_-PpiDΔParv and SurA-His_6_, respectively, as calculated according to [58].

### Analysis of chaperone function *in vitro*

Effects of PpiD proteins on the thermal aggregation of citrate synthase were determined according to [34]. Aggregation was monitored on a Hitachi F-4500 spectrofluorometer with both excitation and emission wavelengths set to 500 nm at a spectral bandwidth of 2.5 nm. Data points were recorded every 0.5 s.

## Authors' contributions

YM analyzed the effect of a *ppiD *deletion and of multicopy *ppiD *on cell envelope phenotypes. BB constructed SurA-depletion strains and performed first depletion experiments. SB designed and conceived the study, conducted the SurA depletion studies, analyzed results and wrote the manuscript. All authors read and approved the final manuscript.

## Supplementary Material

Additional file 1 **Similarity between the N-terminal region of PpiD and the chaperone modules of SurA and Trigger factor (TF)**. (A and B) The N-terminal region of PpiD shows sequence similarity with the N- and C-terminal regions of SurA (A, 25.2% identity) and TF (B, 19.9% identity), respectively. The sequence alignments were generated with CLUSTALW2 [63]. Gray shaded regions indicate the regions of high similarity that were initially identified with LALIGN [64] (31.1% (A) and 24.1% (B) identity, respectively). Identical amino acid residues are indicated by asterisks; conserved and semi-conserved residues are marked with colons and dots, respectively. (C-E) Three-dimensional homology modeling suggests structural similarity of the N-terminal region of PpiD with the chaperone modules of SurA and TF. All structures were visualized in PyMol and are depicted in ribbon representation. (C) Comparative model structure of the N-terminal region of PpiD (red colored) and the N-Ct chaperone module of SurA (blue colored) based on the sequence alignment shown in (A). The model was generated in the Swiss-Model workspace [65] using the structure coordinates of SurA (PDB 1m5y; [42]) as a template. Helices of the N-terminal region of SurA are numbered. A region of PpiD that corresponds to the C-terminal helix ("C helix") of SurA has not yet been identified. (D) Model structure of the N-terminal region of PpiD generated by the automatic program 3D-JIGSAW [66]. (E) Fold of the C-terminal chaperone domain of TF (PDB code 1w26; [41]). The region that shares similarity with PpiD is highlighted in red (corresponding to the gray shaded sequence in (B)). Click here for file

Additional file 2 **Complementation of the growth defect of *ppiD skp surA *cells by wild-type PpiD and its PPIase domain mutants**. Growth of the SurA-depletion strain *P_Llac-O1_*-*surA Δskp ppiD::kan *(SB44961) carrying the empty vector pASK75 or plasmids encoding wild-type proteins and variants of SurA, Skp, and PpiD, respectively. Cells were grown overnight in the presence of IPTG and after dilution spotted on LB plates ± 1 mM IPTG. Plates were incubated at 37°C for 16-24 h. Click here for file

Additional file 3 **Effects of NlpE overproduction in *surA skp *cells**. (A) Growth of the SurA-depletion strains *P_Llac-O1_*-*surA *(SB11019) and *P_Llac-O1_*-*surA Δskp *(SB44997) at 37°C in buffered LB (pH 7.0) with (solid lines) and without (dotted lines) IPTG, resulting in the indicated wild-type (WT), *surA*, *skp *and *surA skp *"genotypes". Strains carried pASK75 (empty vector) or plasmids encoding PpiD and NlpE, respectively. (B) Within the indicated interval (box in panel A) samples were taken and assayed for the activities of σ^E ^and Cpx by monitoring β-galactosidase activity resulting from chromosomal *rpoH*P3*::lacZ *and *cpxP-lacZ *reporter fusions, respectively (see *Methods*). Results represent the average of at least two independent experiments. (C) Western blot detection of SurA in *P_Llac-O1_*-*surA *strains after 265- and 360-minute growth as described in A. Extracts from 4 × 10^7 ^cells were loaded onto each lane. Signal intensities were calculated using Hsc66 as the internal standard for each lane and are shown relative to those in the wild-type strain (rel. Int.). *P_Llac-O1_*-*surA Δskp *cells that carried pASK75 or pNlpE resumed production of SurA after 265-minute growth without IPTG. At about the same time, these cultures also resumed growth (see panel A). The onset of regained SurA production and revived growth varied between growth experiments (data not shown), suggesting that the cultures contained a small population of the cells that was still capable of producing SurA, possibly due to a promoter mutation, and that eventually outgrew the SurA-depleted *Δskp *cell population. In contrast, SurA was hardly detectable during the entire course of growth of PpiD overproducing *surA Δskp *cells. (D) Growth of the strain *P_Llac-O1_*-*surA Δskp *(SB44997) carrying pASK75 or plasmids encoding SurA, PpiD, and NlpE, respectively. Cells were grown overnight in the presence of IPTG, after dilution spotted on LB plates ± 1 mM IPTG, and incubated at 37°C for 16-24 h. Click here for file

Additional file 4 **Effects of *ppiD *and *nlpE *overexpression on the *surA skp *growth and stress response phenotypes**. Table summarizing the levels of suppression of the growth defect and the σ^E ^and Cpx phenotypes of *surA skp *cells caused by multicopy *ppiD *and *nlpE*, respectively. Click here for file
