# Supplementary material for: PpiD is a player in the network of periplasmic chaperones in Escherichia coli
Source: BMC Microbiol. 2010 Sep 29;10:251. doi: 10.1186/1471-2180-10-251 (PMC2956729; doi:10.1186/1471-2180-10-251)

A

```
PpiD   42  AAKVNDQEISRGQFENAFNSERNRMQQQLGDQYSELAANEGYMKTLRQQVLNRLIDEALL
SurA   21  APQVVDKVAAVVNNGVVLESDVDGLMQSVKLNAAQARQQLPDDATLRHQIMERLIMDQII
           *.:* *:  :  :   .::*: : : *.:  : ::   :     ***:*:::*** : ::

PpiD  102  DQYARELKLGISDEQVKQAIFATPAFQVDGKFDNSRYNGILNQMGMTADQYAQALRNQLT
SurA   81  LQMGQKMGVKISDEQLDQAIAN-IAKQNNMTLDQMRSR--LAYDGLNYNTYRNQIRKEMI
            * .::: : *****:.***    * * : .:*: * .  *   *:. : * : :*:::

PpiD  162  TQQLING-VAGTDFMLKGETDELAALVAQQ 190
SurA  138  ISEVRNNEVRRRITILPQEVESLAQQVGNQ 167
            .:: *. *     :*  *.:.**  *.:*
```

B

```
PpiD   43  AKVNDQEISRGQFENAFNSERNRMQQQLGDQYSELAANEGYMKTLRQQVLNRLIDEALLD
TF    295  VKANDIDVPAALIDSEIDVLRRQAAQRFGGN--EKQALELPRELFEEQAKRRVVVGLLLG
           .*.** ::. . ::. ::  *.:  *::*.:  *  * *   : :.:*. .*::   **.

PpiD  103  QYARELKLGISDEQVKQAIFATPAFQVDGKFDNSRYNGILNQMGMTADQYAQALRNQLTT
TF    353  EVIRTNELKADEERVKGLIEEMASAYEDPKEVIEFYS--------KNKELMDNMRNVALE
           :  *  :*  .:*:**  *   .:   * *   . *.        . .:  : :**

PpiD  163  QQLINGVAGTDFMLKGETDELAALVAQQ 191
TF    413  EQAVEAVLAKAKVTEKETTFNELMNQQA 441
           :* ::.* ..  : : **     :  *
```

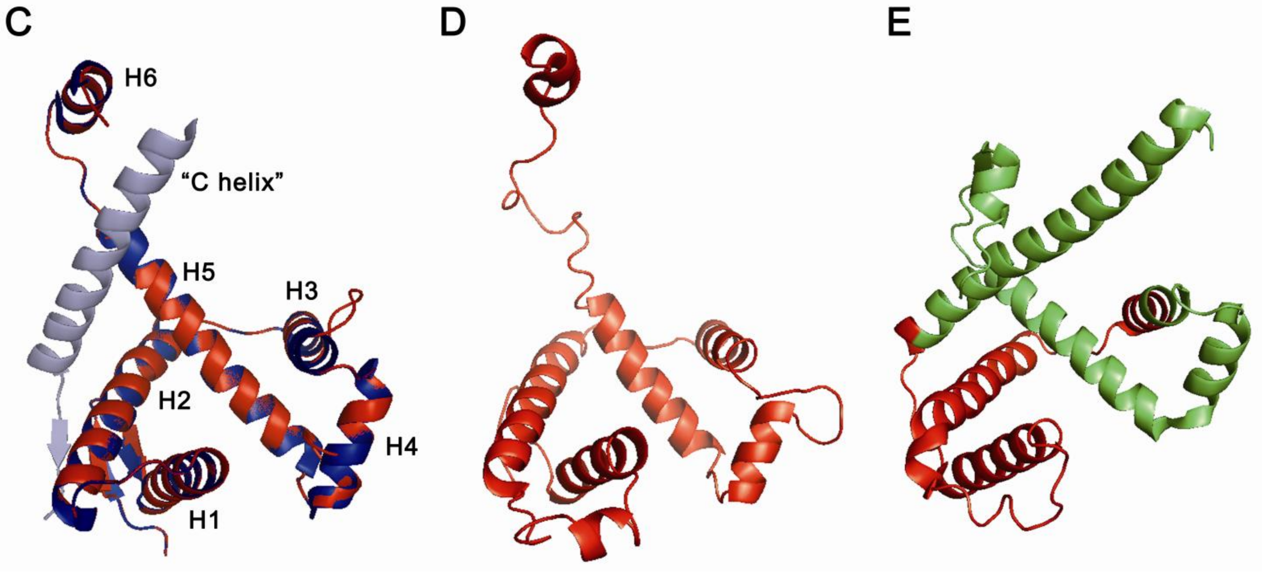

Supplement: Additional file 1 — Similarity between the N-terminal region of PpiD and the chaperone modules of SurA and Trigger factor (TF). (A and B) The N-terminal region of PpiD shows sequence similarity with the N- and C-terminal regions of SurA (A, 25.2% identity) and TF (B, 19.9% identity), respectively. The sequence alignments were generated with CLUSTALW2 [63]. Gray shaded regions indicate the regions of high similarity that were initially identified with LALIGN [64] (31.1% (A) and 24.1% (B) identity, respectively). Identical amino acid residues are indicated by asterisks; conserved and semi-conserved residues are marked with colons and dots, respectively. (C-E) Three-dimensional homology modeling suggests structural similarity of the N-terminal region of PpiD with the chaperone modules of SurA and TF. All structures were visualized in PyMol and are depicted in ribbon representation. (C) Comparative model structure of the N-terminal region of PpiD (red colored) and the N-Ct chaperone module of SurA (blue colored) based on the sequence alignment shown in (A). The model was generated in the Swiss-Model workspace [65] using the structure coordinates of SurA (PDB 1m5y; [42]) as a template. Helices of the N-terminal region of SurA are numbered. A region of PpiD that corresponds to the C-terminal helix ("C helix") of SurA has not yet been identified. (D) Model structure of the N-terminal region of PpiD generated by the automatic program 3D-JIGSAW [66]. (E) Fold of the C-terminal chaperone domain of TF (PDB code 1w26; [41]). The region that shares similarity with PpiD is highlighted in red (corresponding to the gray shaded sequence in (B)). [file 1471-2180-10-251-S1.PDF]
