# Supplementary figures and images for: PpiD is a player in the network of periplasmic chaperones in Escherichia coli
Source: BMC Microbiol. 2010 Sep 29;10:251. doi: 10.1186/1471-2180-10-251 (PMC2956729; doi:10.1186/1471-2180-10-251)

*P<sub>Lac-O1</sub>-surA Δskp ppiD::kan*

1 mM IPTG

no IPTG

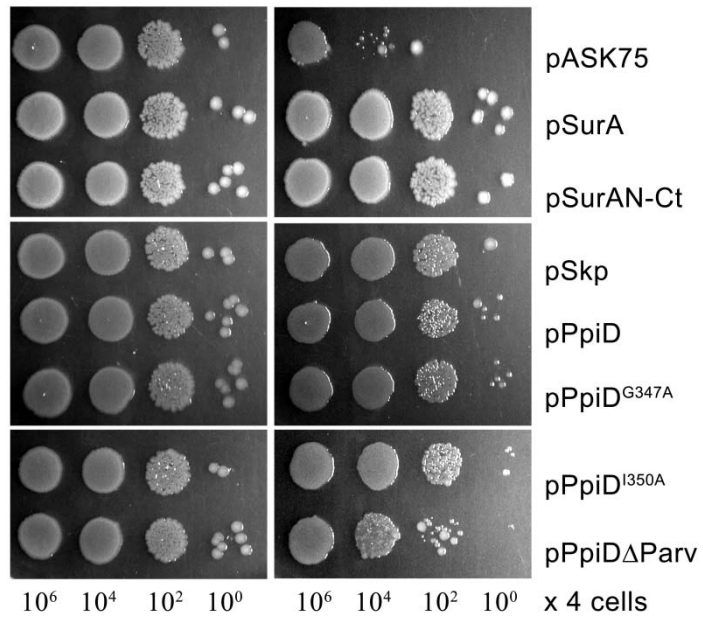

Supplement: Additional file 2 — Complementation of the growth defect of ppiD skp surA cells by wild-type PpiD and its PPIase domain mutants. Growth of the SurA-depletion strain PLlac-O1-surA Δskp ppiD::kan (SB44961) carrying the empty vector pASK75 or plasmids encoding wild-type proteins and variants of SurA, Skp, and PpiD, respectively. Cells were grown overnight in the presence of IPTG and after dilution spotted on LB plates ± 1 mM IPTG. Plates were incubated at 37°C for 16-24 h. [file 1471-2180-10-251-S2.PDF]

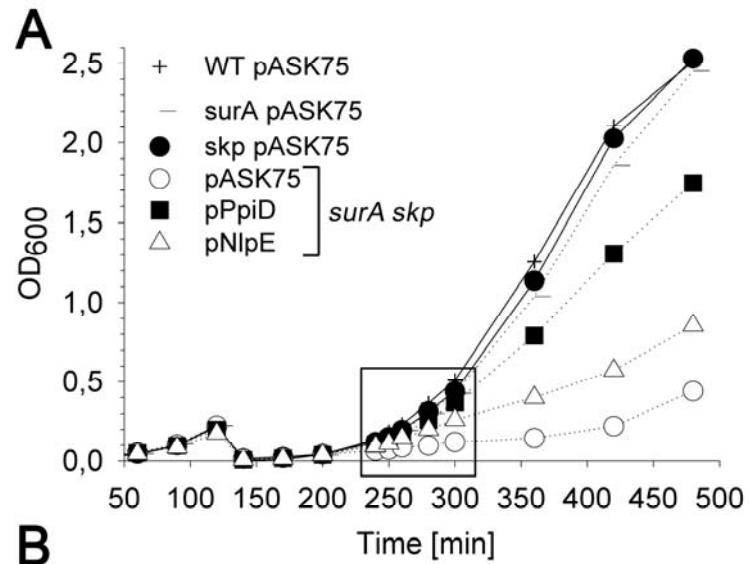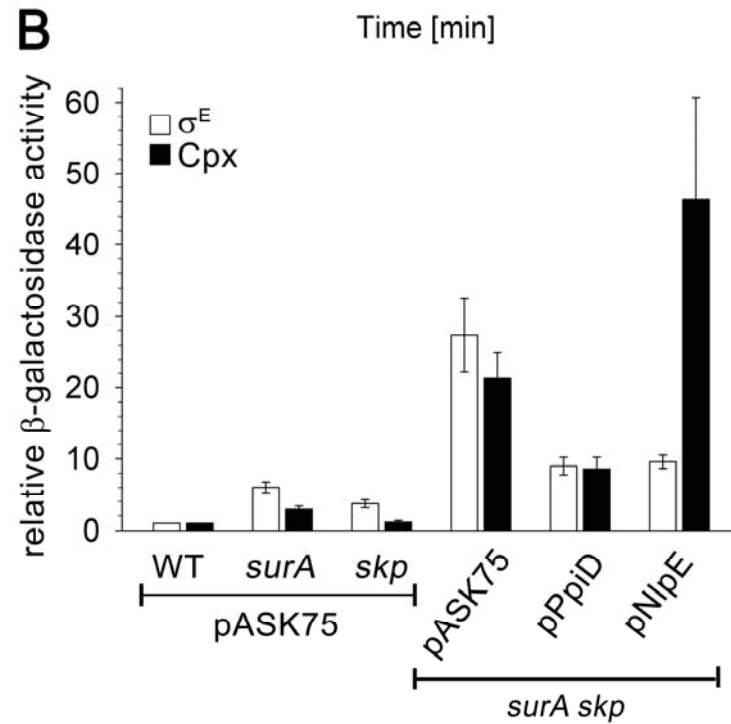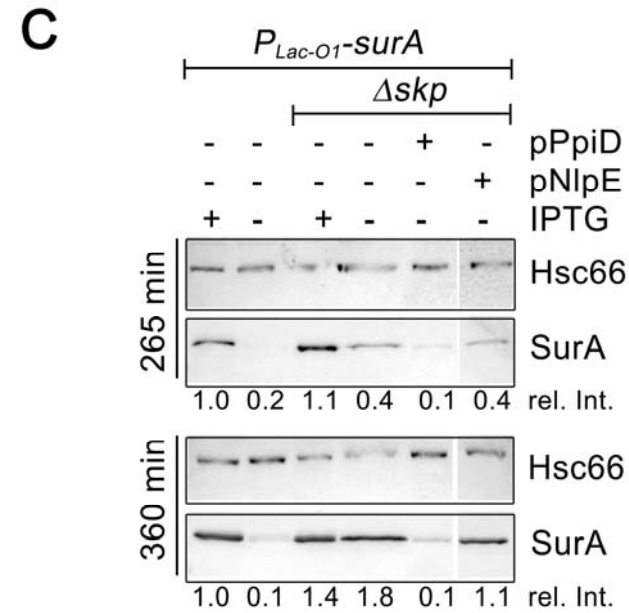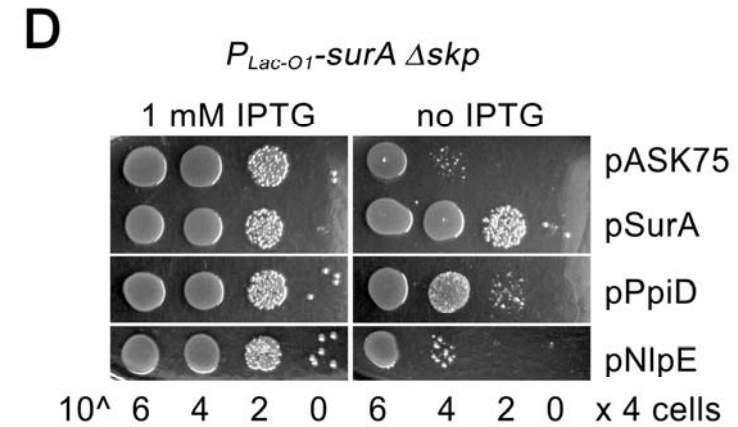

Supplement: Additional file 3 — Effects of NlpE overproduction in surA skp cells. (A) Growth of the SurA-depletion strains PLlac-O1-surA (SB11019) and PLlac-O1-surA Δskp (SB44997) at 37°C in buffered LB (pH 7.0) with (solid lines) and without (dotted lines) IPTG, resulting in the indicated wild-type (WT), surA, skp and surA skp "genotypes". Strains carried pASK75 (empty vector) or plasmids encoding PpiD and NlpE, respectively. (B) Within the indicated interval (box in panel A) samples were taken and assayed for the activities of σE and Cpx by monitoring β-galactosidase activity resulting from chromosomal rpoHP3::lacZ and cpxP-lacZ reporter fusions, respectively (see Methods). Results represent the average of at least two independent experiments. (C) Western blot detection of SurA in PLlac-O1-surA strains after 265- and 360-minute growth as described in A. Extracts from 4 × 10^7^ cells were loaded onto each lane. Signal intensities were calculated using Hsc66 as the internal standard for each lane and are shown relative to those in the wild-type strain (rel. Int.). PLlac-O1-surA Δskp cells that carried pASK75 or pNlpE resumed production of SurA after 265-minute growth without IPTG. At about the same time, these cultures also resumed growth (see panel A). The onset of regained SurA production and revived growth varied between growth experiments (data not shown), suggesting that the cultures contained a small population of the cells that was still capable of producing SurA, possibly due to a promoter mutation, and that eventually outgrew the SurA-depleted Δskp cell population. In contrast, SurA was hardly detectable during the entire course of growth of PpiD overproducing surA Δskp cells. (D) Growth of the strain PLlac-O1-surA Δskp (SB44997) carrying pASK75 or plasmids encoding SurA, PpiD, and NlpE, respectively. Cells were grown overnight in the presence of IPTG, after dilution spotted on LB plates ± 1 mM IPTG, and incubated at 37°C for 16-24 h. [file 1471-2180-10-251-S3.PDF]
