# Supplementary material for: PpiD is a player in the network of periplasmic chaperones in Escherichia coli
Source: BMC Microbiol. 2010 Sep 29;10:251. doi: 10.1186/1471-2180-10-251 (PMC2956729; doi:10.1186/1471-2180-10-251)
Supplement: Additional file 4 — Effects of ppiD and nlpE overexpression on the surA skp growth and stress response phenotypes. Table summarizing the levels of suppression of the growth defect and the σE and Cpx phenotypes of surA skp cells caused by multicopy ppiD and nlpE, respectively. [file 1471-2180-10-251-S4.PDF]

Additional file 4

Effects of *ppiD* and *nlpE* overexpression on the *surA skp* growth and stress response phenotypes

Growth of *surA Δskp* cells carrying the indicated plasmids and levels of induction of the σ^E-dependent and the CpxA/R-regulated envelope stress response pathways in the cells relative to the *surA* single mutant. The minimal and maximal values observed in at least two independent experiments are shown.

**Plasmid**	**Growth**	**σ^E activity** (-fold versus *surA*)	**Cpx activity** (-fold versus *surA*)
pASK75	-	4.3 - 4.8	7.0 - 7.6
pPpiD	+++	1.4 - 1.5	2.8 - 3.1
pPpiDΔParv	++	1.5 - 1.6	4.2 - 4.6
pPpiD(ΔTM)	-	4.7 - 5.7	8.4 - 9.7
pNlpE	(+)[1]	1.4 - 1.5	12.7 - 18.5

[1]weak growth during early exponential phase in liquid LB culture, no growth on solid LB
